# ceas: an R package for Seahorse data analysis and visualization

**DOI:** 10.1093/bioinformatics/btae503

**Published:** 2024-08-12

**Authors:** Rachel (Rae) J House, James P Eapen, Hui Shen, Carrie R Graveel, Matthew R Steensma

**Affiliations:** Department of Cell Biology, Van Andel Research Institute, Grand Rapids, MI 49503, United States; Department of Metabolism and Nutritional Programming, Van Andel Research Institute, Grand Rapids, MI 49503, United States; Department of Epigenetics, Van Andel Research Institute, Grand Rapids, MI 49503, United States; Department of Epigenetics, Van Andel Research Institute, Grand Rapids, MI 49503, United States; Department of Cell Biology, Van Andel Research Institute, Grand Rapids, MI 49503, United States; Department of Cell Biology, Van Andel Research Institute, Grand Rapids, MI 49503, United States; Helen DeVos Children’s Hospital, Corewell Health, Grand Rapids, MI 49503, United States; College of Human Medicine, Michigan State University, Grand Rapids, MI 49503, United States; Orthopedic Oncology, Corewell Health, Grand Rapids, MI 49503, United States

## Abstract

**Summary:**

Measuring cellular energetics is essential to understanding a matrix’s (e.g. cell, tissue, or biofluid) metabolic state. The Agilent Seahorse machine is a common method to measure real-time cellular energetics, but existing analysis tools are highly manual or lack functionality. The Cellular Energetics Analysis Software (*ceas*) R package fills this analytical gap by providing modular and automated Seahorse data analysis and visualization.

**Availability and implementation:**

*ceas* is available on CRAN (https://cran.r-project.org/package=ceas). Source code and installable tarballs are freely available for download at https://github.com/jamespeapen/ceas/releases/ under the MIT license. Package documentation may be found at https://jamespeapen.github.io/ceas/. *ceas* is implemented in R and is supported on macOS, Windows and Linux.

## 1 Introduction

In 1929, Otto Warburg demonstrated that cancer cells favor aerobic glycolysis over oxidative phosphorylation, an energetic phenomenon termed the “Warburg effect” (Ward and Thompson 2012). Since Warburg’s seminal publication, numerous studies have demonstrated that energetic changes underscore cancer growth and contribute to metabolic reprogramming, a cellular state characterized by elevated macromolecular synthesis (Hanahan and Weinberg 2011, Ward and Thompson 2012, Boroughs and DeBerardinis 2015). Given the biological importance of energetic processes, measuring real-time energetics is essential for understanding the metabolic state of a given matrix (e.g. cell, tissue, or biofluid).

The Agilent Seahorse machine is one of the most common methods of measuring real-time cellular energetics (Gu *et al.* 2021). The Seahorse has two primary readouts, oxygen consumption rate (OCR) and extracellular acidification rate (ECAR), that enable inference of a matrix’s oxidative phosphorylation (OXPHOS) and glycolytic rates.

**Figure 1.**
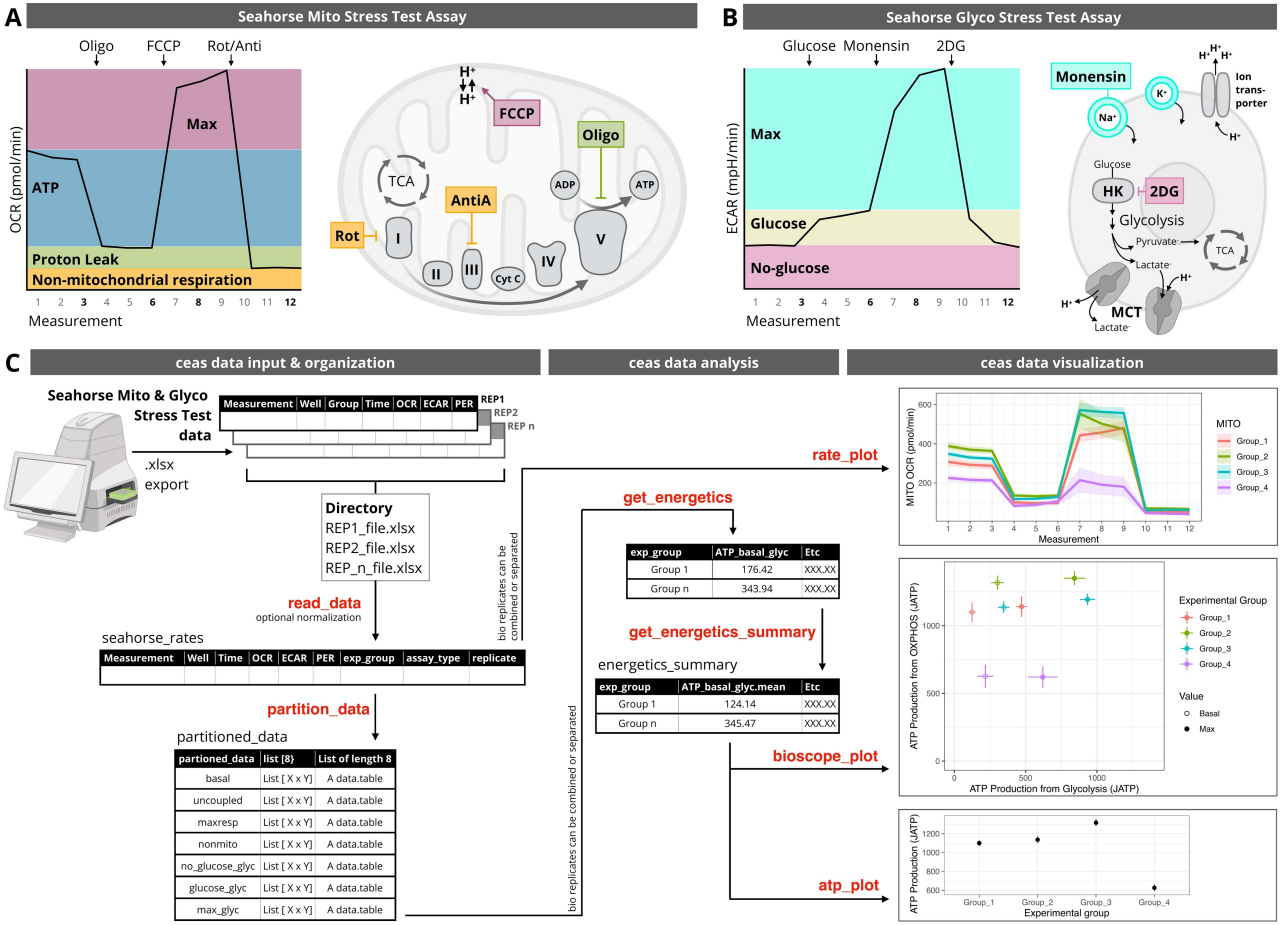
*ceas* enables modular, automated Seahorse data analysis and visualization. (A) Seahorse Mito Stress Test assay and inhibitor schematic: Oligomycin (oligo), Fluoro-carbonyl cyanide phenylhydrazone (FCCP), rotenone (rot), and antimycin (anti) are mitochondrial-directed energetic inhibitors. (B) Seahorse Glyco Stress Test assay and compound schematic. Glucose, monensin, and 2-deoxy-d-glucose (2DG) are glycolysis-directed energetic compounds. (C) *ceas* R package workflow schematic.

OCR is a measurement of media oxygen flux. OCR serves as a proxy of OXPHOS because O_2_ is the final electron acceptor in the electron transport chain (ETC) (Mookerjee *et al.* 2017). Since multiple cellular processes consume oxygen, the Mito Stress Test assay uses the following inhibitors to strategically modulate cellular oxygen consumption: oligomycin (Oligo), fluoro-carbonyl cyanide phenylhydrazone (FCCP), and rotenone and antimycin A (Rot/AntiA) (Brand and Nicholls 2011). Oligomycin is a ETC complex V inhibitor that inhibits ATP-linked OCR (Fig. 1A; Gu *et al.* 2021). FCCP is an uncoupling agent that allows unimpeded electron flow across the mitochondrial inner membrane and induces maximal (“Max”) experimental OCR (Fig. 1A). Rotenone and antimycin A are ETC complex I and complex III inhibitors, respectively. Together, rotenone and antimycin A inhibit mitochondria-related OCR (Fig. 1A; Gu *et al.* 2021).

ECAR is a measurement of media acidification and a proxy of multiple acidifying processes, notably glycolysis (Mookerjee *et al.* 2015). Glycolysis produces pyruvate, which can be converted to lactate by lactate dehydrogenase (LDH). The LDH reaction strongly favors the production of lactate, resulting in lactate export and cellular efflux via monocarboxylate transporters (MCTs) (Rogatzki *et al.* 2015). MCT transport requires the input of a hydrogen ion (H^+^), so lactate efflux exports H^+^ into the extracellular environment (Fig. 1B). The Seahorse machine measures media H^+^ content, from which it calculates ECAR. Many non-glycolytic cellular reactions produce H^+^. To account for this, the Seahorse Glyco Stress Test assay uses the following compounds to strategically modulate media acidification: glucose, monensin, and 2-deoxy-d-glucose (2DG) (Fig. 1B). In addition, to control for tricarboxylic acid (TCA) cycle CO_2_-derived acidification, oxidative acidification is subtracted from total acidification (Mookerjee *et al.* 2015). Glucose is the primary substrate for glycolysis, so glucose addition stimulates “Basal” glycolytic ECAR. Acidification prior to glucose addition can be attributed to non-glycolytic sources or residual media glucose. Monensin is an ionophore that indirectly enhances glycolysis by preventing the “collapse of membrane ion gradients” (Mookerjee *et al.* 2016), so monensin treatment induces maximal (“Max”) ECAR (Gu *et al.* 2021). The ceas variable, “Max,” represents the maximal, experimental ECAR. To confirm that an experimental maximal is the biological maximum, a Glycolytic Capacity Assay must be performed (Mookerjee *et al.* 2016). Lastly, 2DG inhibits the glycolytic enzyme, hexokinase, so 2DG addition inhibits glycolysis-related ECAR (Gu *et al.* 2021).

To compare OCR (pmol/min) and ECAR (mpH/min), their units must be converted to a shared metric. Mookerjee *et al.* provided the mathematical foundation for OCR and ECAR conversion to joules of ATP produced (JATP), enabling inference of oxidative and glycolytic ATP production (Mookerjee *et al.* 2017). Since Mookerjee’s publication, no tools have been developed to make this foundational computation scriptable and automated (Supplementary Table S1). Existing and published R-based tools include *OCRbayes* (Zhang *et al.* 2021), *OCR-stats* (Yépez *et al.* 2018), and *sybilxf* (Ramirez *et al.* 2017). *OCRbayes* provides a Bayesian hierarchical modeling framework for OCR analysis. *OCR-stats* performs inter-replicate OCR statistical analyses and outlier identification. *sybilxf* integrates Seahorse energetic data with genome-scale metabolic modeling to predict substrate flux. However, none of these cited R tools enable JATP conversions. Existing non-R tools include Agilent’s Windows-based report generators and Seahorse Analytics web application, which perform JATP conversions but lack scriptability and flexibility. The Cellular Energetics Analysis Software (*ceas*) R package fills these analytical gaps by providing modular and automated Seahorse OCR and ECAR analysis, JATP calculation, and data visualization.

## 2 Implementation

*ceas* is an R package that requires R version 4.1.0 or newer. All data manipulation is backed by the data.table library (https://rdatatable.gitlab.io/data.table/) for fast operations. The *ceas* workflow can be divided into three general categories: data input and organization, data analysis, and data visualization (Fig. 1C). *ceas* is modular, allowing data extraction for user-directed statistical analysis and visualization at any point during the default *ceas* workflow. Generated plots are similarly extensible as they are generated using *ggplot2* (Wickham 2016). Of note, *ceas* default conditions are set to analyze both Mito and Glyco Stress Test assay data, but *ceas* functions can accommodate multiple input assays, including Mito Stress Test data and data in the configurations of Mookerjee *et al.* (2017) and Ma *et al.* (2019) (Supplemental Fig. S1). All functions are documented on the package website along with vignettes describing the analysis workflow.

### 2.1 *ceas* data input and organization

The *ceas* data import function, read_data, enables import and integration of multiple biological replicates and/or multiple assay types from a common directory and also enables optional data normalization according to cell number or micrograms of protein. Previous work has reported high inter-plate variation (Yépez *et al.* 2018), so ceas allows users to estimate summary statistics across technical replicates (intra-file, via ordinary least squares) or across biological replicates [inter-file, via either ordinary least squares or linear mixed-effects models (Bates *et al.* 2015)]. Since data import uses the *readxl* (https://readxl.tidyverse.org/) package and does not require Microsoft Excel or a graphical user interface, *ceas* can be run on text-based high-performance computers. The Seahorse Wave software requires a user-defined “Group” for each experimental sample. When the Wave file is exported, this user-defined “Group” identifier is organized as a “Group” column. The ceas read_data function requires a standardized “Group” column that contains both (i) experimental group and (ii) assay type (e.g. MITO or GLYCO), separated by a space (e.g. “Group_1 MITO” or “Group_2 GLYCO”). Of note, the number of experimental groups is user-defined and theoretically unlimited. This standardized input format allows the read_data function to correctly infer the experimental group (exp_group) and subset the data by assay type (assay_type). While existing datasets will need to be modified to this format, future Wave experiment files can be designed to fit the *ceas* import format. It is worth nothing, that while *ceas* was designed for Seahorse XF96 data analysis, any Wave output matching the XF96 format (e.g. Agilent HS Mini, tested) is compatible with *ceas*. Following data import and optional normalization, the partition_data function subsets data based on the “Measurements” column. These assay measurement time points correspond to specific energetic states relevant for future calculations (see bolded “Measurement” numbers in Fig. 1A and B). As with other analysis tools, the utility of *ceas* output is predicated on experimentally robust input data.

### 2.2 *ceas* data analysis

After Seahorse data is partitioned according to user-defined values, the get_energetics function calculates inferred basal and maximal JATP produced from OXPHOS and glycolysis according to calculations published by Mookerjee *et al.* (2017) (Supplementary Fig. S2). The get_energetics_summary function returns mean, standard deviation (SD), and confidence intervals (CI, default = 95%) of the calculated JATP values. Calculations are available for export if custom visualization is required.

### 2.3 *ceas* data visualization

*ceas* can generate three data visualizations: OCR and ECAR plots, “bioenergetic scope” plots, and ATP plots. The rate_plot function provides an overview of OCR or ECAR over time and enables cross-group energetic comparisons before and after the addition of energetics-modulating compounds. The rate plot lines represent mean group OCR or ECAR over sequential Measurement time points and the shaded variance region represents SD or CI according to user choice. The bioscope_plot function plots a 2D representation of bioenergetic scope by the experimental groups. Bioenergetic scope describes the theoretical energetic space in which a matrix operates. The bioenergetic scope coordinates are JATP from OXPHOS on the *y*-axis and JATP from glycolysis on the *x*-axis. The points represent mean basal and maximal JATP from OXPHOS and glycolysis, and the vertical and horizontal lines represent the SD or CI of JATP from OXPHOS or glycolysis, respectively. Lastly, the atp_plot function plots group JATP values, which enables cross-group OXPHOS and glycolytic JATP comparisons at basal and maximal conditions. The ATP plot points represent the mean basal or max JATP from OXPHOS or glycolysis, and the crossbar boundaries represent the SD or CI of JATP.

## 3 Application

To showcase *ceas* functionality, Seahorse Mito and Glyco Stress Test assays were performed on four novel MCF7-dervied (ATCC, Cat#HTB-22) cell lines—Group_1 (control), Group_2 (mutant A), Group_3 (mutant B), and Group_4 (mutant C). Assays were run on a Seahorse XF96 Analyser.

For Seahorse analysis, cells were grown in Dulbecco’s modified eagle medium (DMEM, Gibco, Cat#21063029), 10% fetal bovine serum (Corning, Lot#13721001), 5 ml penicillin-streptomycin (Gibco, Cat#15070063) and treated with 10 nM beta-estradiol (Sigma, Cat#E2758) for 48 h. Cells were then trypsinized, collected, quenched, and spun down. Cells were counted using a BioRad cell counter and plated in poly-d-lysine-coated (Gibco, Cat#A3890401) Seahorse assay plates (Agilent, Cat#103794-100) at 30 000 cells per well. Cells were spun down and allowed to fully adhere for 4 h at 37°C in a 5% CO_2_ incubator. After 4 h, the growth media was gently removed by pipetting, and 180 µl Seahorse XF DMEM media (Agilent, Cat#103575-100) was added. Mito Stress Test XF DMEM assay media contained 25 mM glucose (Fischer Scientific, Cat#D16-500), 4 mM glutamine (Sigma, Cat#25030-081), and 1 mM pyruvate (Gibco, Cat#11360-070). Glyco Stress Test XF DMEM assay media contained 4 mM glutamine. XF DMEM media has a pH of 7.4, a pK_1_ of 6.093 at 37°C, and a buffering factor of 0.1 (mpH/pmol H^+^) (Mookerjee *et al.* 2015). All assays were performed according to Agilent Mito Stress Test and Glyco Stress Test instructions.

Mito and Glyco Stress Test Wave files were exported in the Microsoft Excel format and run through all ceas functions. ceas rate_plot output illustrated that Group_4 has decreased OCR (pmol/min) and Group_2 and Group_3 have increased ECAR (mpH/min) compared to the Group_1 control line (Supplementary Fig. S3A). ceas bioscope_plot output demonstrated that Group_4 OXPHOS ATP production is half that of the Group_1 control (Supplementary Fig. S3B). The bioscope_plot output also illustrated that Group_2 and Group_3 cells have, overall, larger bioenergetic scopes, indicative of greater energetic flexibility (Supplementary Fig. S3B) (Mookerjee *et al.* 2017). The atp_plot illustrated that all mutant cell lines have increased ATP production from glycolysis compared to the Group_1 control (Supplementary Fig. S3C). In conclusion, *ceas* identified bioenergetic differences across four, novel, MCF7-derived cell lines and provided information about each cell line’s metabolic state.

## 4 Summary

*ceas* enables modular, automated Seahorse data analysis and visualization. Because the Agilent Seahorse machine is a common method of measuring real-time cellular energetics, *ceas* applicability extends across research disciplines, from basic to translational research. *ceas* is freely available to install from GitHub and CRAN.

## Supplementary Material

btae503_Supplementary_Data

## Data Availability

The data underlying this article are available in the *ceas* repository at https://github.com/jamespeapen/ceas and can be accessed under the “inst/extdata” directory. Code used to generate the figures may be found in the package vignette: https://jamespeapen.github.io/ceas/articles/ceas.html.

## References

[btae503-B1] Bates D , Mächler M, Bolker B et al Fitting linear mixed-effects models using lme4. J Stat Soft 2015;67:1–48.

[btae503-B2] Boroughs LK , DeBerardinis RJ. Metabolic pathways promoting cancer cell survival and growth. Nat Cell Biol 2015;17:351–9. 25774832 10.1038/ncb3124 PMC4939711

[btae503-B3] Brand MD , Nicholls DG. Assessing mitochondrial dysfunction in cells. Biochem J 2011;435:297–312. 21726199 10.1042/BJ20110162 PMC3076726

[btae503-B4] Gu X , Ma Y, Liu Y et al Measurement of mitochondrial respiration in adherent cells by seahorse XF96 cell mito stress test. STAR Protoc 2021;2:100245. 33458707 10.1016/j.xpro.2020.100245 PMC7797920

[btae503-B5] Hanahan D , Weinberg RA. Hallmarks of cancer: the next generation. Cell 2011;144:646–74. 21376230 10.1016/j.cell.2011.02.013

[btae503-B6] Ma EH , Verway MJ, Johnson RM et al Metabolic profiling using stable isotope tracing reveals distinct patterns of glucose utilization by physiologically activated CD8+ T cells. Immunity 2019;51:856–70.e5. 31747582 10.1016/j.immuni.2019.09.003

[btae503-B7] Mookerjee SA , Gerencser AA, Nicholls DG et al Quantifying intracellular rates of glycolytic and oxidative ATP production and consumption using extracellular flux measurements. J Biol Chem 2017;292:7189–207. 28270511 10.1074/jbc.M116.774471 PMC5409486

[btae503-B8] Mookerjee SA , Goncalves RLS, Gerencser AA et al The contributions of respiration and glycolysis to extracellular acid production. Biochim Biophys Acta 2015;1847:171–81. 25449966 10.1016/j.bbabio.2014.10.005

[btae503-B9] Mookerjee SA , Nicholls DG, Brand MD et al Determining maximum glycolytic capacity using extracellular flux measurements. PLoS One 2016;11:e0152016. 27031845 10.1371/journal.pone.0152016 PMC4816457

[btae503-B10] Ramirez AK , Lynes MD, Shamsi F et al Integrating extracellular flux measurements and genome-scale modeling reveals differences between brown and white adipocytes. Cell Rep 2017;21:3040–8. 29241534 10.1016/j.celrep.2017.11.065 PMC5841536

[btae503-B11] Rogatzki MJ , Ferguson BS, Goodwin ML et al Lactate is always the end product of glycolysis. Front Neurosci 2015;9:22. 25774123 10.3389/fnins.2015.00022 PMC4343186

[btae503-B12] Ward PS , Thompson CB. Metabolic reprogramming: a cancer hallmark even Warburg did not anticipate. Cancer Cell 2012;21:297–308. 22439925 10.1016/j.ccr.2012.02.014 PMC3311998

[btae503-B13] Wickham H. ggplot2: Elegant Graphics for Data Analysis. New York: Springer-Verlag; 2016.

[btae503-B14] Yépez VA , Kremer LS, Iuso A et al OCR-Stats: robust estimation and statistical testing of mitochondrial respiration activities using Seahorse XF Analyzer. PLoS One 2018;13:e0199938. 29995917 10.1371/journal.pone.0199938 PMC6040740

[btae503-B15] Zhang X , Yuan T, Keijer J et al OCRbayes: a Bayesian hierarchical modeling framework for Seahorse extracellular flux oxygen consumption rate data analysis. PLoS One 2021;16:e0253926. 34265000 10.1371/journal.pone.0253926 PMC8282019

