# Supplementary material for: ceas: an R package for Seahorse data analysis and visualization
Source: Bioinformatics. 2024 Aug 12;40(8):btae503. doi: 10.1093/bioinformatics/btae503 (PMC11349193; doi:10.1093/bioinformatics/btae503)
Supplement: btae503_Supplementary_Data [file btae503_supplementary_data.zip › House_Eapen_supplementary_file_03.pdf]

Supplementary Figure 1

A ceas default assay design: Mito and Glyco Stress Test assays

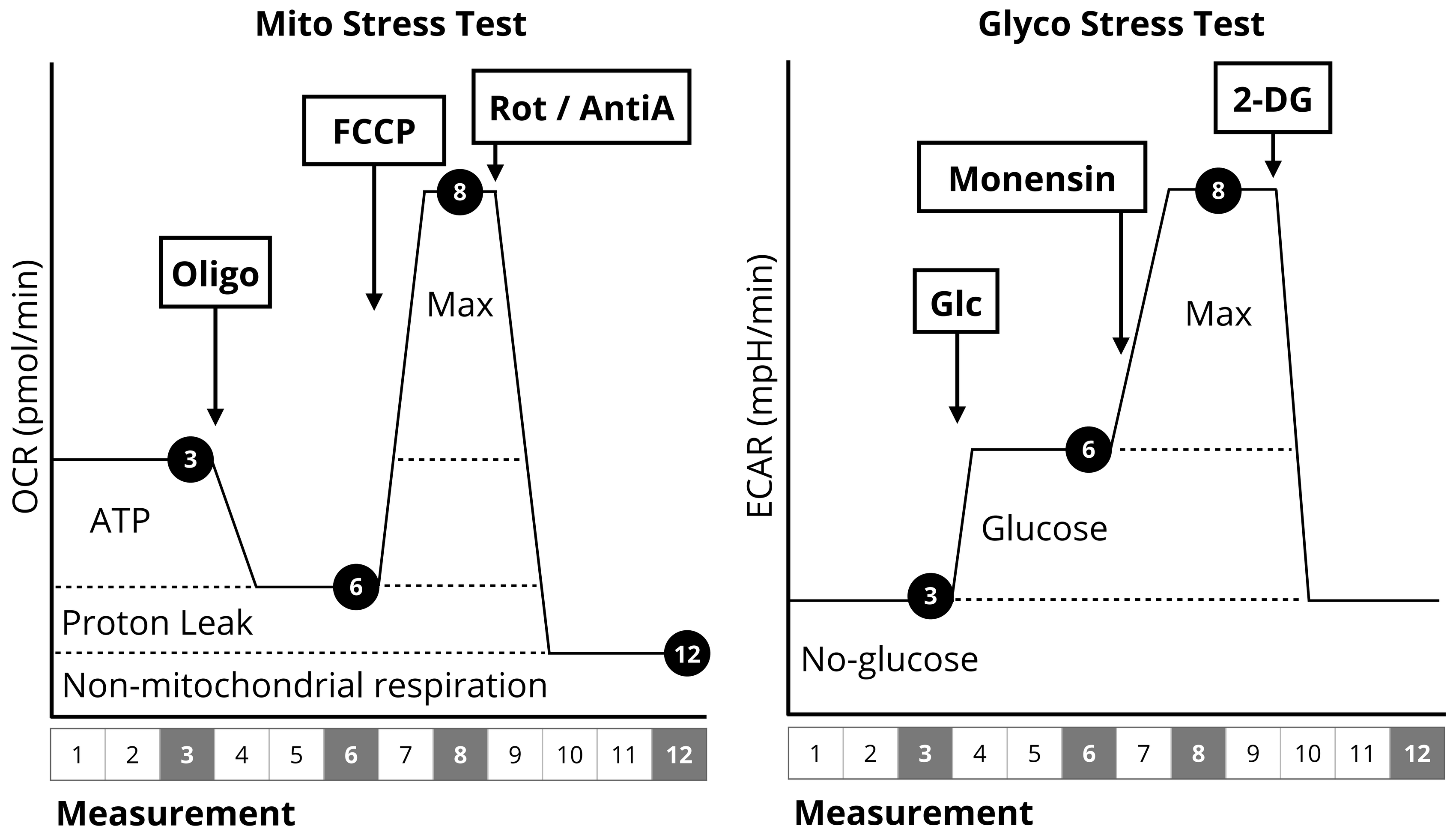


```
partitioned_data <- partition_data(seahorse_rates,
  assay_types = list(
    basal = "MITO",
    uncoupled = "MITO",
    maxresp = “MITO",
    nonmito = "MITO",
    no_glucose_glyc = "GLYCO",
    glucose_glyc = "GLYCO",
    max_glyc = "GLYCO"
  ),
  basal_tp = 3,
  uncoupled_tp = 6,
  maxresp_tp = 8,
  nonmito_tp = 12,
  no_glucose_glyc_tp = 3,
  glucose_glyc_tp = 6,
  max_glyc_tp = 8
)
```

B Mookerjee et al. 2017 assay design

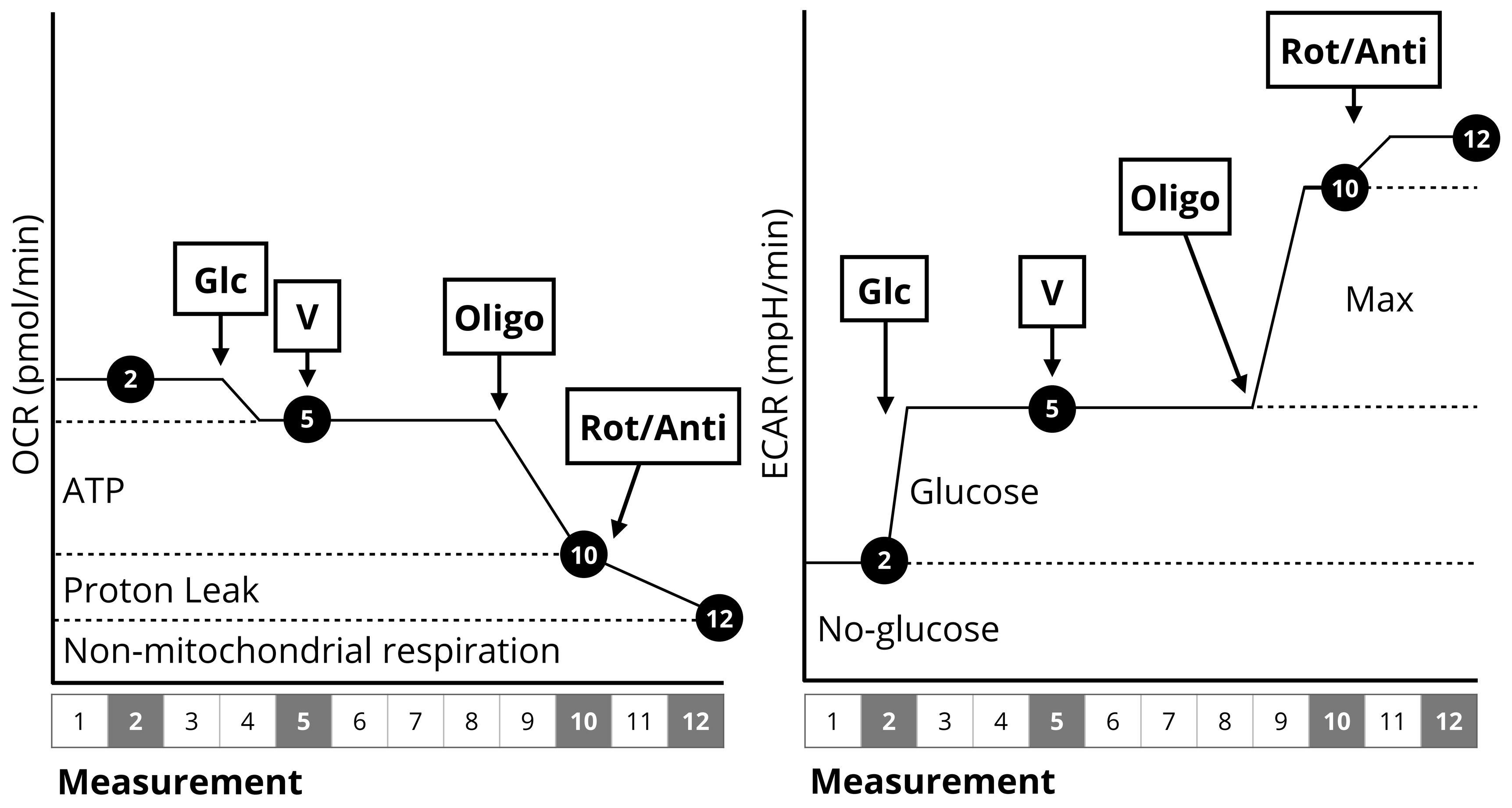


```
partitioned_data <- partition_data(seahorse_rates,
  assay_types = list(
    basal = "RefAssay",
    uncoupled = "RefAssay",
    maxresp = NA,
    nonmito = "RefAssay",
    no_glucose_glyc = "RefAssay",
    glucose_glyc = "RefAssay",
    max_glyc = NA
  ),
  basal_tp = 5,
  uncoupled_tp = 10,
  nonmito_tp = 12,
  maxresp = NA,
  no_glucose_glyc_tp = 1,
  glucose_glyc_tp = 5,
  max_glyc = NA
)
```

C Modified Mito Stress Test assay (Ma et al. 2019)

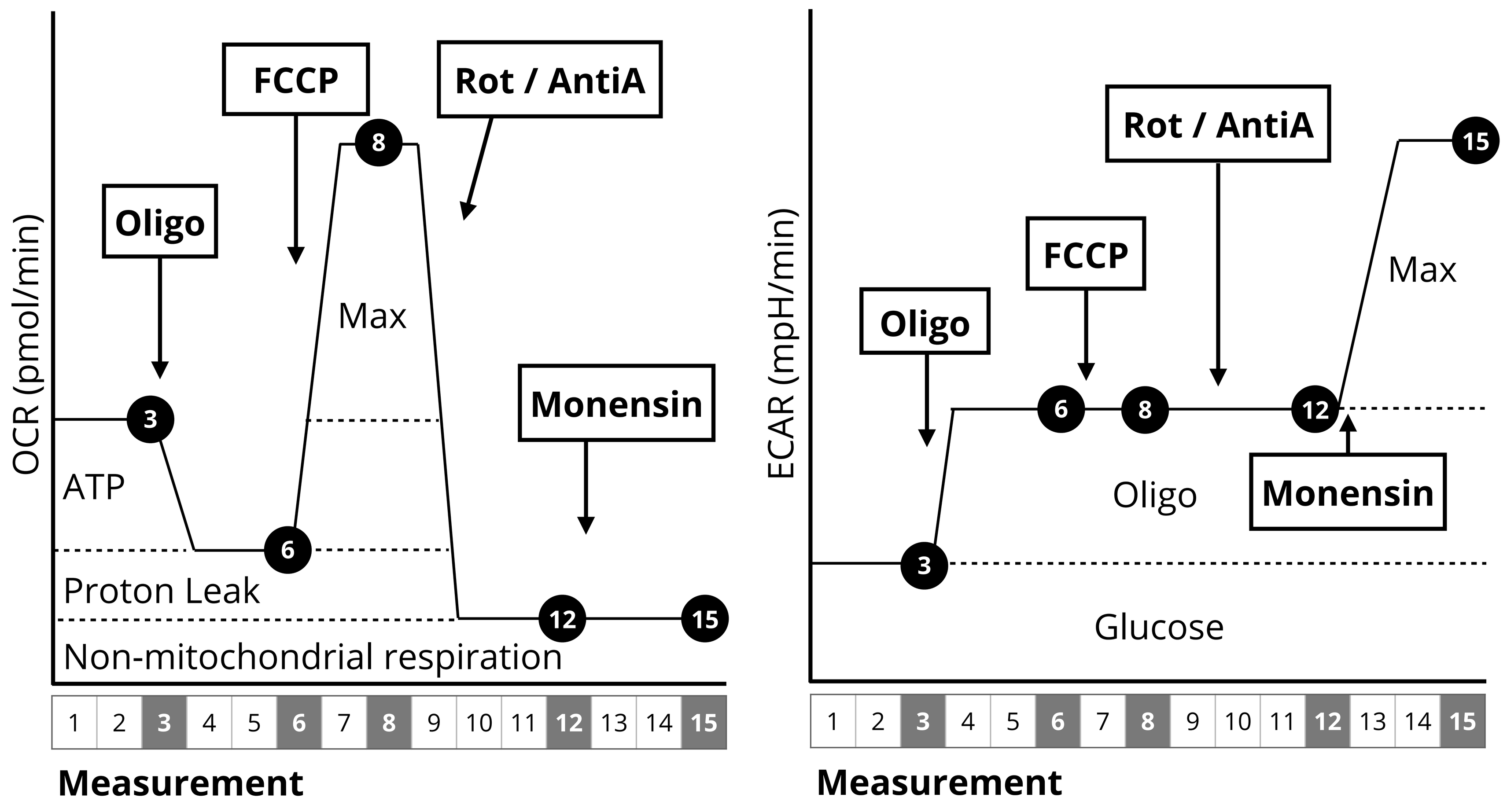


```
partitioned_data <- partition_data(seahorse_rates,
  assay_types = list(
    basal = "MITO",
    uncoupled = "MITO",
    maxresp = “MITO",
    nonmito = "MITO",
    no_glucose_glyc = NA,
    glucose_glyc = "MITO",
    max_glyc = "MITO"
  ),
  basal_tp = 3,
  uncoupled_tp = 6,
  maxresp_tp = 8,
  nonmito_tp = 12,
  no_glucose_glyc_tp = NA,
  glucose_glyc_tp = 3,
  max_glyc_tp = 15
)
```

Supplementary Figure 1. Example ceas-compatible assay designs and their respective partition_data codes. A) Mito and Glyco Stress Test assay schematics and partition_data function information. B) Mookerjee et al. 2017 assay schematic and partition_data function information. C) Example Modified Mito Stress Test assay schematic and partition_data function information (Ma et al. 2019).

Supplementary Figure 2

A

Mookerjee et al. assay design | **ceas default assay design: Mito and Glyco Stress Tests**

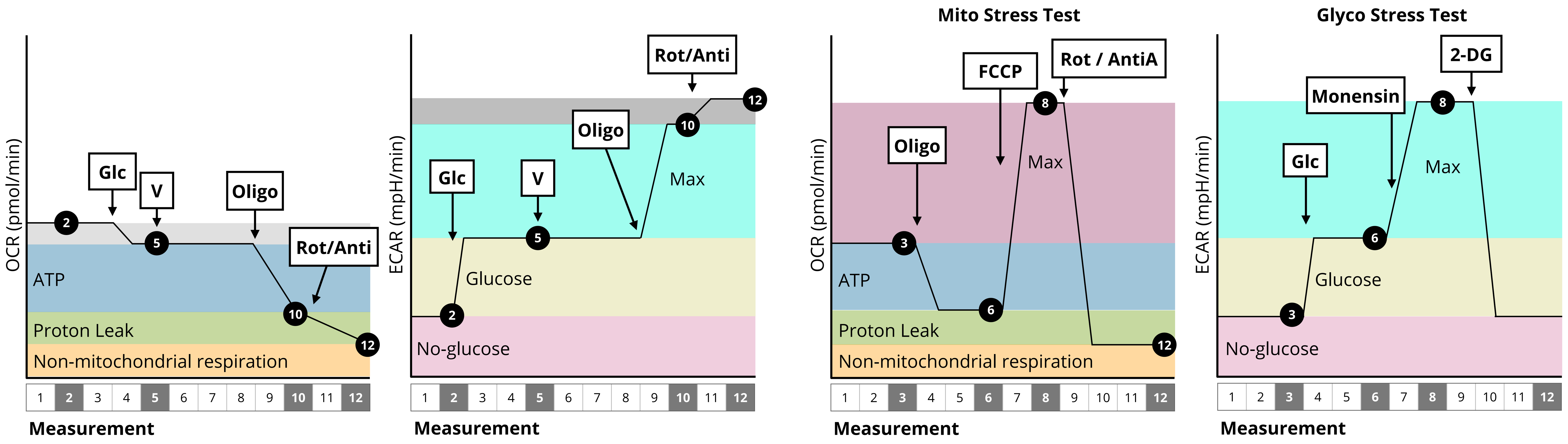

Mookerjee et al.

Variable	Biological metric	Measurement
basal	Basal respiration (no glucose)	2
glucose	Respiration (with glucose)	5
oligo	ATP-coupled respiration	10
rot/anti	Non-mitochondrial respiration	12

Variable	Value (in KRPH media)
pH	7.4
buffer (mpH/pmol H+)	0.045
pKa	6.093
max H+/O2	1
ATP/lactate	1
P/O_{glyc}	0.167
P/O_{oxphos}	2.486
P/O_{tca}	0.121

ceas

Variable	Biological metric	Assay	Measurement
basal	Basal respiration	MITO	3
uncoupled	ATP-coupled respiration	MITO	6
maxresp	Maximal ("Max") experimental respiration	MITO	8
nonmito	Non-mitochondrial respiration	MITO	12
no_glucose_glyc	No glucose added acidification	GLYCO	3
glucose_glyc	Glucose-associated acidification	GLYCO	6
max_glyc	Maximal ("Max") experimental acidification	GLYCO	8

Variable	Value (in XF DMEM)
pH	7.4
buffer (mpH /pmol H+)	0.1
pKa	6.093
max H+/O2	1
ATP/ lactate	1
P/O_{glyc}	0.167
P/O_{oxphos}	2.486
P/O_{tca}	0.121

B

Mookerjee Equation #1

$$OCR_{mito} = OCR_{total} - OCR_{rot/anti}$$

Mookerjee et al.

Experimental Variable	Calculation	
OCR_{mito} (basal)	basal$OCR	- rot/anti$OCR
OCR_{mito} (glucose)	glucose$OCR	- rot/anti$OCR
OCR_{mito} (oligomycin)	oligo$OCR	- rot/anti$OCR

ceas

Experimental Variable	Calculation	
basal_mito_resp	basal$OCR	- nonmito$OCR
uncoupled_mito_resp	uncoupled$OCR	- nonmito$OCR
max_mito_resp	maxresp$OCR	- nonmito$OCR

C

Mookerjee Equation #2

$$OCR_{coupled} = (OCR_{total} - OCR_{oligo}) / 0.908 \text{ (i.e. hyperpolarization factor)}$$

Mookerjee et al.

Experimental Variable	Calculation			
$OCR_{coupled}$ (basal)	(basal$OCR	- oligo$OCR)	/	0.908
$OCR_{coupled}$ (glucose)	(glucose$OCR	- oligo$OCR)	/	0.908
$OCR_{coupled}$ (oligomycin) (~0)	(oligo$OCR	- oligo$OCR)	/	0.908

ceas

Experimental Variable	Calculation
coupled_mito_resp	(basal_mito_resp - uncoupled_mito_resp) x 0.908

D

Mookerjee Equation #3

$$PPR_{total} = ECAR_{total} / \text{buffer (+mito acidification)}$$

Mookerjee et al.

Experimental Variable	Calculation		
$ECAR_{total}$ (basal)	basal$ECAR		
$ECAR_{total}$ (glucose)	glucose$ECAR		
$ECAR_{total}$ (oligomycin)	oligo$ECAR		
PPR_{total} (basal)	$ECAR_{total}$ (basal)	/ buffer	(+mito)
PPR_{total} (glucose)	$ECAR_{total}$ (glc)	/ buffer	(+mito)
PPR_{total} (oligomycin)	$ECAR_{total}$ (oligo)	/ buffer	(+mito)

ceas

Experimental Variable	Calculation		
no_glucose_glyc_acidification	no_glucose_glyc$ECAR		
glucose_glyc_acidification	glucose_glyc$ECAR		
max_glyc_acidification	max_glyc$ECAR		
ppr_basal	glucose_glyc_ acidification	/ buffer	(+mito)
ppr_max	max_glyc_ acidification	/ buffer	(+mito)

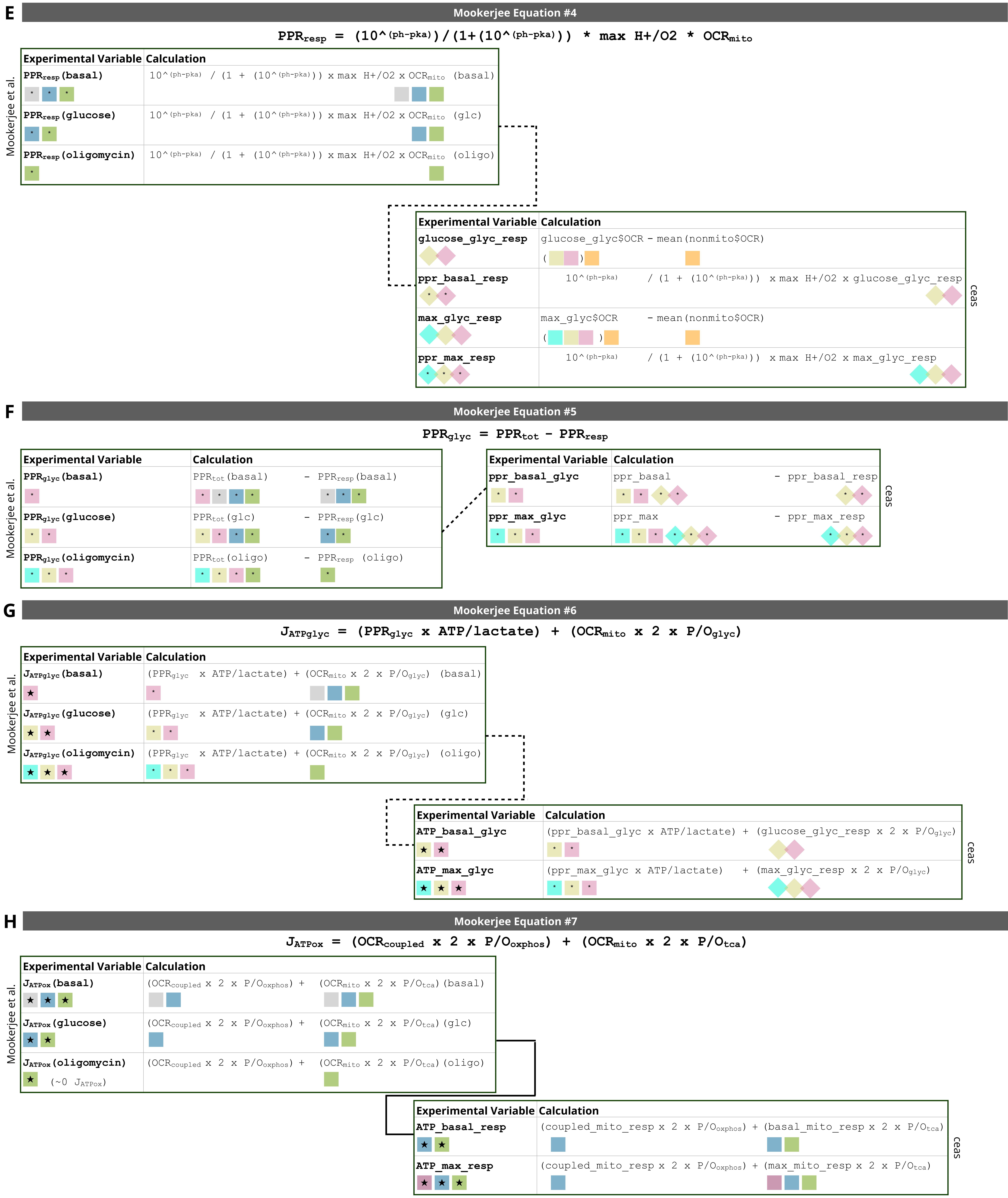

Supplementary Figure 2. Schematic comparison of ceas vs. Mookerjee et al. 2017 calculations and subsetting. A) CEAS vs. Mookerjee assay design and subsetting references. B) ceas vs. Mookerjee equation #1 calculations. C) ceas vs. Mookerjee equation #2 calculations. D) ceas vs. Mookerjee equation #3 calculations. E) ceas vs. Mookerjee equation #4 calculations. F) ceas vs. Mookerjee e equation #5 calculations. G) ceas vs. Mookerjee equation #6 calculations. H) ceas vs. Mookerjee equation #7 calculations.

Supplementary Figure 3

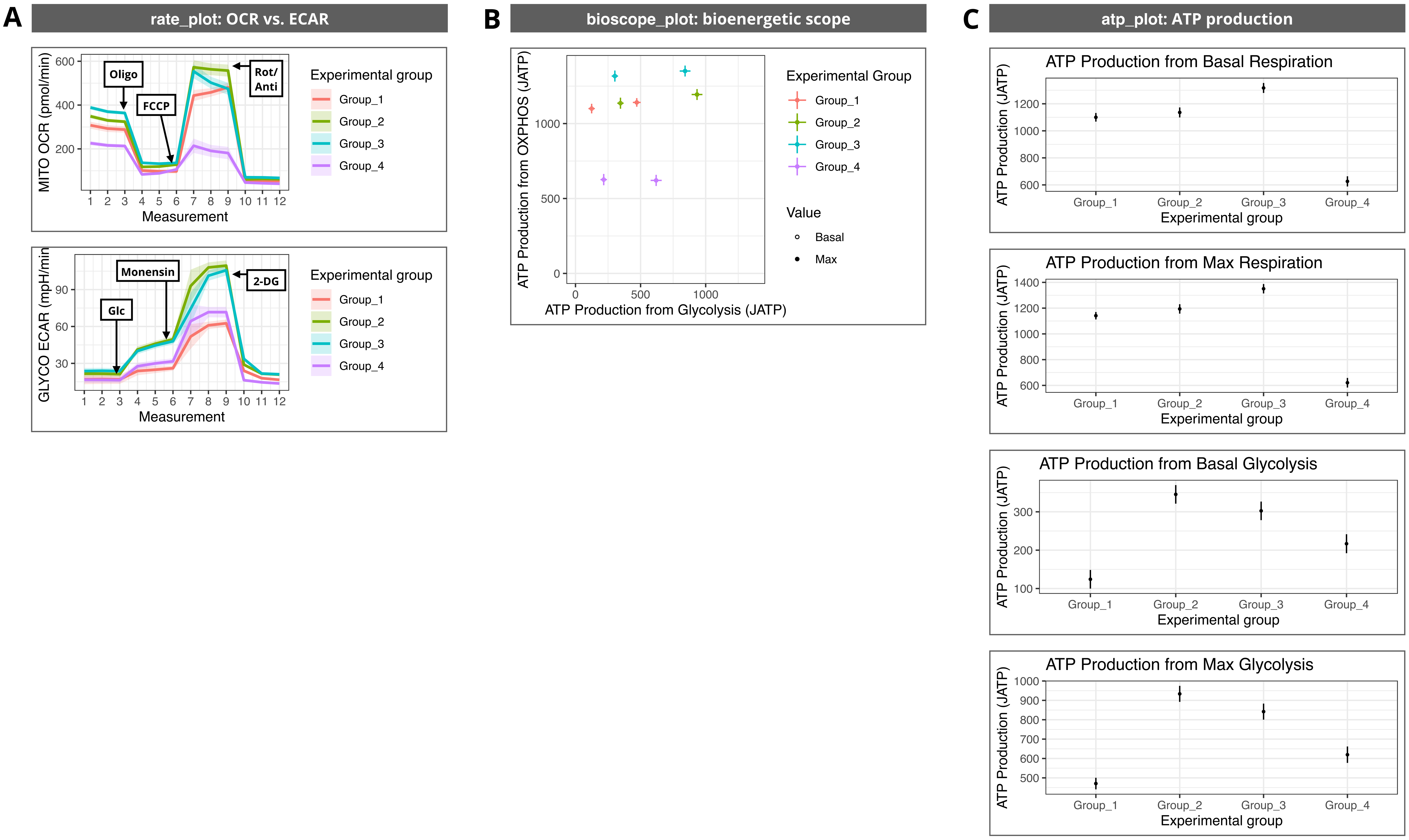

Supplementary Figure 3. The Group_1 (control), Group_2 (mutant A), Group_3 (mutant B), and Group_4 (mutant C) MCF7-derived cell lines have unique bioenergetic profiles. A) OCR (pmol/min) and ECAR (mpH/min) per experimental group during the Mito and Glyco Stress Tests, respectively. The lines represent the geometric means, and the shaded regions represent the 95% confidence interval (model="mixed"). n = 2, 12 technical replicates per biological replicate. B) Bioenergetic scope plot. The points represent mean basal and maximal ATP from OXPHOS and glycolysis, and the vertical and horizontal lines represent the 95% confidence interval (model="mixed") of JATP from OXPHOS or glycolysis, respectively. n = 2, 12 technical replicates per biological replicate. C) ATP production at basal and maximal OXPHOS and glycolysis across experimental groups. The points represent the geometric mean, and the crossbar boundaries represent the 95% confidence interval (model="mixed"). n = 2, 12 technical replicates per biological replicate.

Supplementary Table 1

Software	Seahorse compatibility	Software type	Operating system	Input format	Scriptable	JATP calculation	Analyzed metric (OCR/ECAR)	Replicate files supported	Input assay	Modular	Packaged
Agilent report generators†	XF24, HS Mini, XF96	Windows executable	Windows only	Wave file	No	Yes	OCR and ECAR	No	Mutiple	Yes	Yes
Agilent multi-file XF report generator†	XF24, HS Mini, XF96	Windows executable	Windows only	Wave file	No	Yes	OCR and ECAR	Yes*	Mutiple	Yes	Yes
Agilent Seahorse Analytics	XF24, HS Mini, XF96	Web application	Windows, MacOS, Linux	Wave file	No	Yes	OCR and ECAR	Yes*	Mutiple	Yes	Yes
ceas	XF96 (tested), HS Mini (tested), XF24 (putative)	R package	Windows, MacOS, Linux	Wave excel file	Yes	Yes	OCR and ECAR	Yes	Mutiple	Yes	Yes
Mookerjee	XF96	Excel spreadsheet	Windows, MacOS, Linux	Manual data input	No	Yes	OCR and ECAR	No	Mookerjee et. al (2017)	No	NA
OCRbayes	XF24 and XF96	R package	Windows, MacOS, Linux	Wave excel file	Yes	No	OCR	No	Mito Stress Test	Yes	No
OCR-stats	XF24 and XF96	R package	Windows, MacOS, Linux	Wave excel file	Yes	No	OCR and ECAR	No	Mito Stress Test	Yes	No
rSeahorse¶	XF24	R package	Windows, MacOS, Linux	Wave excel file	Yes	Yes	OCR	No	Mutiple	Yes	Yes
sybilxf	XF96	R package	Windows, MacOS, Linux	Wave excel file	Yes	No	OCR and ECAR	Yes	Mutiple	Yes	Yes

Supplementary Table 1. Comparison of existing Seahorse analysis and visualization tools to ceas. *if assays were run on the same XF machine and with identical plate layouts. † Requires purchase of Microsoft Office on Windows and MacOS or LibreOffice calc on Linux. Software from Agilent, is closed-source so we could not find information on maintenance status. ¶rSeahorse is available at https://github.com/whzemuch/rSeahorse
